# Kinetic and Potentiometric Characteristics of Ferredoxin: NADP^+^ Oxidoreductase from *Chlorobaculum tepidum*

**DOI:** 10.3390/ijms27010481

**Published:** 2026-01-02

**Authors:** Dominykas Laibakojis, Daisuke Seo, Narimantas Čėnas, Mindaugas Lesanavičius

**Affiliations:** 1 Department of Xenobiotics Biochemistry, Institute of Biochemistry, Life Sciences Center, Vilnius University, Saulėtekio Av. 7, LT-10257 Vilnius, Lithuania; dominykas.laibakojis@chgf.stud.vu.lt (D.L.); mindaugas.lesanavicius@gmc.vu.lt (M.L.); 2 Division of Material Sciences, Graduate School of Natural Science and Technology, Kanazawa University, Kakuma, Kanazawa 920-1192, Japan; dseo@se.kanazawa-u.ac.jp

**Keywords:** quinones, nitroaromatics, flavins, redox cycling, ferredoxin: NADP^+^ oxidoreductase, single-electron reduction, flavosemiquinone

## Abstract

*Chlorobaculum tepidum* ferredoxin: ${\mathrm{NADP}}^{+}$ oxidoreductase (*Ct*FNR) is a dimeric thioredoxin reductase (TrxR)-type FNR, whose mechanism and redox properties are poorly characterized. In this work, we focused on the reoxidation mechanisms of its flavin adenine dinucleotide (FAD) cofactor using quinones (Q), nitroaromatics (${\mathrm{ArNO}}_{2}$), and other nonphysiological oxidants with different single-electron reduction midpoint potentials ($E_{7}^{1}$) and electrostatic charge. Like in other FNRs, the rate-limiting step of the reaction is the reoxidation of FAD semiquinone (${\mathrm{FADH}}^{•}$). However, only one FAD per dimer functions in *Ct*FNR due to some nonequivalence of the NADP(H) binding domains in separate subunits. The reactivity of Q increases with increasing $E_{7}^{1}$, while ${\mathrm{ArNO}}_{2}$ form another analogous series of lower reactivity. The compounds are reduced in a dominant single-electron way. These data are consistent with an “outer sphere” electron transfer mechanism. On the basis of reactions with 3-acetylpyridine adenine dinucleotide phosphate, the two-electron reduction midpoint potential of FAD at pH 7.0 is −0.282 V. In *Ct*FNR, 11% ${\mathrm{FADH}}^{•}$ was stabilized at equilibrium. Calculated electron transfer distances in reactions with Q and ${\mathrm{ArNO}}_{2}$ were in the range of 2.6–3.4 Å. Taken together with previous studies of *Rhodopseudomonas palustris* and *Bacillus subtilis* FNRs, this work allows us to generalize the information on the catalytic ant thermodynamic properties of TrxR-type FNRs. In addition, our data may be valuable from an applied perspective, e.g., the use of redox mediators in photobioelectrochemical systems or microbial cells based on anoxygenic phototrophic bacteria.

## 1. Introduction

Ferredoxin: ${\mathrm{NADP}}^{+}$ oxidoreductases (FNRs, E.C. 1.18.1.2) transform a two-electron transfer into a single-electron one, and according to flavoenzyme classification belong to the class of dehydrogenases–electrontransferases [1,2]. A typical feature of FNRs is the relative high stability of a neutral flavin adenine dinucleotide semiquinone (${\mathrm{FADH}}^{•}$) intermediate, which enables efficient redox reactions between two molecules of ferredoxin (Fd) and NADP(H). FNRs are found in all domains of life and function in versatile cellular metabolic processes. In oxygenic photosynthesis, plant-type FNRs reduce ${\mathrm{NADP}}^{+}$ at the expense of two molecules of reduced Fd generated by an FeS-type photoreduction center photosystem I, thus providing NADPH for ${\mathrm{CO}}_{2}$ assimilation (Equation (1)) [3,4,5]. In nonphotosynthetic processes, FNRs reduce Fd by NADPH, with reduced Fd then being used for nitrate assimilation or the synthesis of cell membrane components [5,6,7]. In the case of iron deficiency low redox potential electron carrier flavodoxin (Fld) can function as the physiological partner of FNRs in place of Fd [8]. FNRs and their protein redox partners are characterized by electrostatic and hydrophobic interactions [9].

(1) $$2{\mathrm{Fd}}_{\mathrm{red}}+{\mathrm{NADP}}^{+}+H^{+}⇌2{\mathrm{Fd}}_{\mathrm{ox}}+\mathrm{NADPH}$$

**Figure 1:**
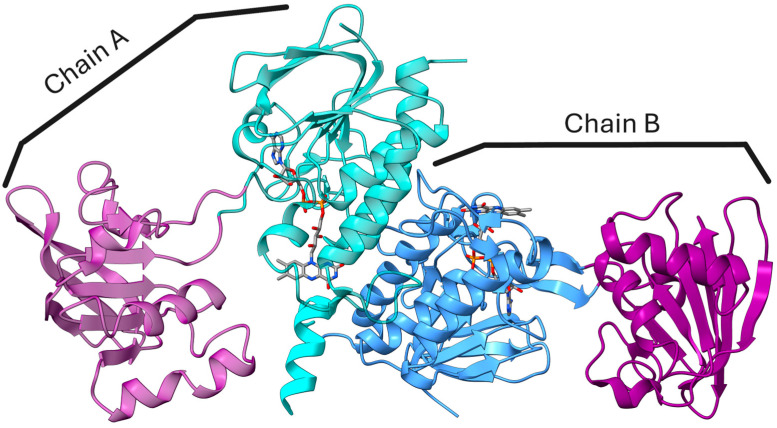
The 3D structure of the asymmetric homodimeric *Ct*FNR (PDB ID: 3AB1). FAD-binding domain is colored teal and NADPH binding domain is colored purple for chain A. Chain B FAD-binding domain is colored blue, NADPH binding domain is colored purple and C-terminal subdomain is colored teal. Enzyme-bound FADs are shown and colored according to elements. The molecular graphics were made using UCSF ChimeraX (version 1.7) [13].

FNR from the thermophilic green sulfur bacterium *Chlorobaculum tepidum* (*Ct*FNR) belongs to a relatively poorly studied group of thioredoxin reductase (TrxR)-type FNRs [10,11]. The distinguishing features of this type FNRs are a homodimeric structure and a FAD-binding domain comprising two discontinuous segments separated by an NADP(H)-binding domain (Figure 1) just as found in disulfide reductases [5]. In the case of *Ct*FNR, the FAD-binding domain consists of amino acid residues 13–131 and 262–329, while residues 134–258 build up an NADP(H) binding domain [12]. The distance between the isoalloxazine ring of FAD and nicotinamide ring of ${\mathrm{NADP}}^{+}$ is assumed to be approximately 15 Å in the crystal structure, too great for an efficient hydride transfer (Figure 1) [12]. The two nucleotide binding domains are connected by flexible hinge regions allowing for domain rotation during catalysis. As in other TrxR-type FNRs, there are also π–π stacking interactions between the isoalloxazine ring of FAD and Tyr57 on the *si*-side and Phe337 on its *re*-side [12]. Another notable peculiarity of *Ct*FNR is the asymmetric arrangement of the homodimer, i.e., NADP(H) domain in one protomer is in “open” conformation, while the equivalent domain in the other protomer is “relatively closed” in the crystal structure. The asymmetry may be related to the presence of the distinct long hydrophilic C-terminal extension which acts as a subdomain to physically tether one protomer of the dimer while interacting with the other protomer [12].

As an obligate photoautotrophic anaerobe, *C. tepidum* utilizes the reverse tricarboxylic acid cycle for ${\mathrm{CO}}_{2}$ fixation and this pathway needs a continuous supply of low-potential reductants, e.g., reduced Fd [14,15,16]. *C. tepidum* utilizes an FeS-type photoreaction center as a sole photosystem for photosynthesis which reduces Fds directly as just the case of photosystem I in oxygenic photosynthesis [15,17,18]. Although *C. tepidum* FNR can catalyze the reduction of ${\mathrm{NAD}(P)}^{+}$ in the presence of photosynthetic reaction center and Fd from *C. tepidum* [11], kinetic details of the interactions of Fds with *Ct*FNR have not been studied [17,19]. The positively charged domain of Lys60, Lys262 and Arg321 is suggested to be candidate for Fd binding based on the crystal structure [12].

While kinetic studies of *Ct*FNR with electron donors/acceptors such as Fd are scarce, the reactions with NADPH and ${\mathrm{NADP}}^{+}$ are studied with steady state and pre-steady state approaches [18,20]. At pH 7.0, the initial phase of reduction of the enzyme by excess NADPH is characterized by *k ≥* 500 $s^{-1};$ however, after 500 ms, the FAD absorbance decrease is about 50% of that expected during the complete *Ct*FNR reduction. Similarly, reoxidation of dithionite-reduced *Ct*FNR by ${\mathrm{NADP}}^{+}$ restores only about 70% of the absorption of oxidized FAD. This is likely related to the above-mentioned structural nonequivalence of its subunits.

In our previous studies, we determined the mechanisms of reactions of homologous TrxR-type FNRs from *Rhodopseudomonas palustris* (*Rp*FNR) and *Bacillus subtilis* (*Bs*FNR) with nonphysiological oxidants quinones (Q), nitroaromatic compounds (${\mathrm{ArNO}}_{2}$), and inorganic complexes [21,22]. This type of reaction can reveal properties of redox proteins that are important for their reactions with physiological redox partners in solution, such as electrostatic or hydrophobic interaction, electron transfer mechanisms, etc., and is valuable for comparative analysis [23]. In many cases, especially when it comes to the reduction of quinones and nitroaromatics by flavoenzymes, this information can also elucidate the mechanisms of the therapeutic/toxic effects or biodegradation pathways of these compounds [24,25,26]. Moreover, FNRs in general have been recently investigated for various applications, e.g., for NADPH regeneration [27,28], and microbial production of ethanol or biofuels [29,30].

In this work we studied the reactions of *Ct*FNR with a panel of nonphysiological oxidants, mainly quinones and nitroaromatics with a wide range of oxidative potency and different electrostatic charge. Taken together with the determination of the redox potentials of the first and second electron transfer of the FAD cofactor of this enzyme, these data provide valuable comparative information, further generalizing the catalytic mechanisms of TrxR-type FNRs.

## 2. Results

### 2.1. Presteady-State Kinetics Studies of CtFNR Reoxidation

**Figure 2:**
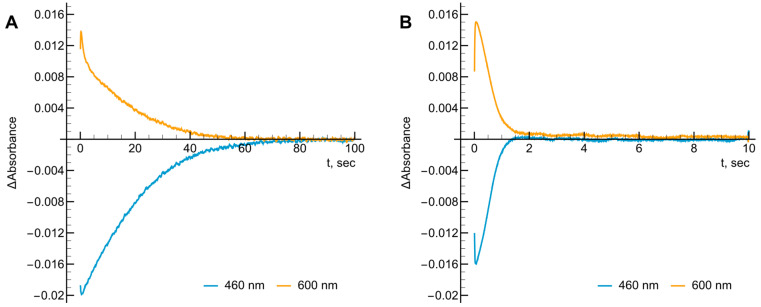
The absorbance changes at 460 and 600 nm during the reduction of 5 μM *Ct*FNR with 50 μM NADPH and its subsequent reoxidation by $O_{2}$ (**A**) or 250 μM duroquinone (**B**). Concentrations reported after mixing.

Existing evidence suggests that FAD reduction in the dimeric *Ct*FNR by NADPH may be partial [18,20]. Thus, we examined the possible manifestation of this phenomenon in the kinetics of *Ct*FNR reoxidation under multiple turnover conditions by tetramethyl-1,4-benzoquinone (duroquinone, DQ). We utilized DQ as an electron acceptor due to its optical transparency above 460 nm and its semiquinone being rapidly reoxidized by oxygen. The control experiment performed without DQ shows very fast reduction of FAD by NADPH followed by its slow reoxidation by $O_{2}$, evidenced by a slow reappearance of absorbance at 460 nm, and a corresponding initial absorbance increase with a slow decrease at 600 nm (Figure 2A). The addition of DQ increases the rate of reoxidation approximately 40-fold with spectral changes at both wavelengths being on the same timescale (Figure 2B). Most importantly, the maximum degree of FAD reduction determined from the difference in absorbance between oxidized and dithionite-reduced *Ct*FNR ($\Delta \varepsilon_{460}\;=\;9.6\;{\mathrm{mM}}^{-1}{\mathrm{cm}}^{-1}$ [18]) was equal to 46.4% ($O_{2}$), and 33.1% (DQ) which is 2–2.5 times less than in the case of *R. palustris* or *B. subtillis* FNR, when the studies were performed with the same NADPH and DQ concentrations [21,22]. These findings imply that only a fraction of the FAD cofactor participates in the catalytic turnover of *Ct*FNR. However, for convenience, the rate constants presented later will be calculated based on the total enzyme concentration.

The rate constants of enzyme reoxidation ($k_{\mathrm{ox}}$) under multiple turnover conditions were calculated using Equation (2) [31]:

(2) $$k_{\mathrm{ox}}=\frac{{\left[\mathrm{NADPH}\right]}_{0}}{{\left[E_{\mathrm{red}}\right]}_{\max}\times t_{1/2\left(\mathrm{off}\right)}}$$

**Figure 3:**
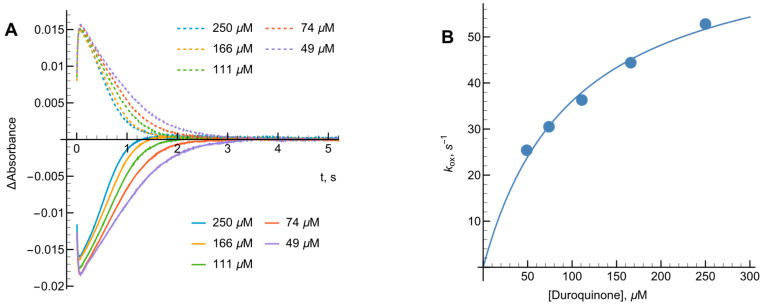
(**A**) The kinetics of *Ct*FNR (5 μM) reduction and reoxidation under multiple turnover conditions with a varying concentration of duroquinone in the presence of 50 μM NADPH followed at 460 nm (solid curves) and 600 nm (dashed curves). (**B**) Michaelis–Menten fit for the dependence of reoxidation constant $k_{\mathrm{ox}}$ on the concentration of duroquinone. Values obtained from (**A**) upon treatment according to Equation (2).

In this expression, ${\left[\mathrm{NADPH}\right]}_{0}$ represents the starting NADPH concentration, while ${\left[E_{\mathrm{red}}\right]}_{\max}$ denotes the peak level of reduced enzyme observed during turnover. The term $t_{1/2\left(\mathrm{off}\right)}$ refers to the duration measured between the points where the reduced enzyme reaches half its maximal concentration during both the formation and decay phases. Based on the data in Figure 2A, the $k_{\mathrm{ox}}$ for oxygen was 1.8 $s^{-1}$. By varying DQ concentrations (Figure 3A,B) we calculated $k_{\mathrm{ox}}$ of 73.2 ± 4.6 $s^{-1}$ at infinite oxidant concentration and the apparent bimolecular oxidation rate constant was determined to be 8.0 ± 1.4 × $10^{5}$ $M^{-1}s^{-1}$.

### 2.2. Steady-State Kinetics and Oxidant Substrate Specificity of CtFNR

**Figure 4:**
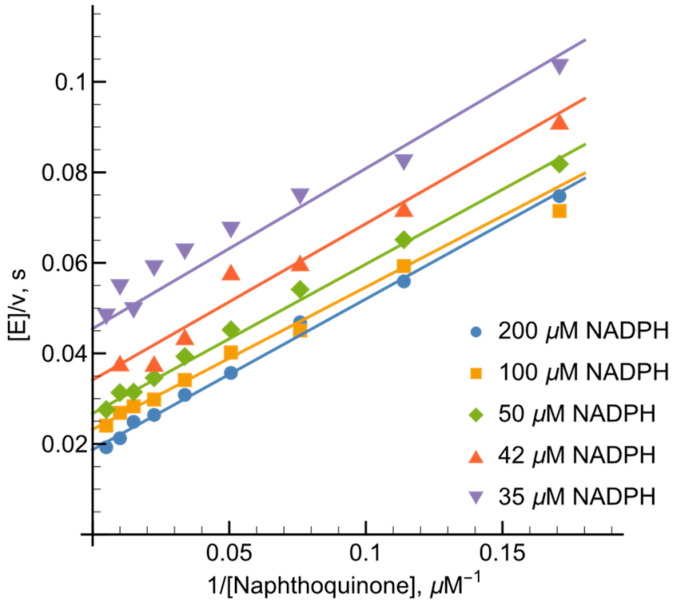
Lineweaver–Burk plots of the steady-state kinetics of the oxidation of NADPH catalyzed by *Ct*FNR with varied concentrations of 1,4-naphthoquinone (NQ) under constant concentrations of NADPH.

At NADPH concentrations ranging from 20 to 200 µM, NADPH–oxidase activity of *Ct*FNR was determined to be 2.1 $s^{-1}$, a value consistent with our presteady-state kinetic observations (Figure 2A). The $k_{\mathrm{cat}}$ values reported herein have been adjusted to account for this baseline oxidase activity. Previous research [21,22] has identified 1,4-naphthoquinone derivatives as effective nonphysiological oxidants for FNR enzymes. In our study, varying the concentrations of both NADPH and 1,4-naphthoquinone yielded a series of parallel lines in double-reciprocal (Lineweaver–Burk) plots (Figure 4). This kinetic pattern strongly suggests that *Ct*FNR operates via a “ping-pong” catalytic mechanism, where the enzyme alternates between its oxidized and reduced forms without forming a ternary complex.

Calculated according to Equation (11) (see Section 4), the $k_{\mathrm{cat}}$ for 1,4-naphthoquinone reduction at saturating NADPH concentrations was 80.9 ± 10.3 $s^{-1}$. The bimolecular rate constants ($k_{\mathrm{cat}}/K_{m}$) for 1,4-napthoquinone and NADPH were determined to be 3.3 ± 0.4 × $10^{6}$ $M^{-1}s^{-1}$ and 9.3 ± 1.3 × $10^{5}$ $M^{-1}s^{-1}$, respectively.

**Figure 5:**
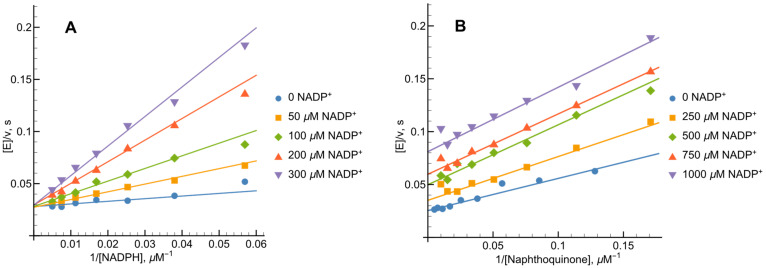
Inhibition of *Ct*FNR-catalyzed reactions by reaction product ${\mathrm{NADP}}^{+}$. (**A**) Competitive inhibition at varied concentrations of NADPH in the presence of 100 μM 1,4-naphthoquinone. (**B**). Uncompetitive inhibition at varied 1,4-naphthoquinone concentrations in the presence of 100 μM NADPH.

We observed that ${\mathrm{NADP}}^{+}$ acts as a product inhibitor of the *Ct*FNR-catalyzed quinone reduction. Specifically, at a fixed 1,4-napthoquinone concentration (100 μM) ${\mathrm{NADP}}^{+}$ exhibited competitive inhibition relative to NADPH (Figure 5A) with $K_{ic}$ = 33.8 ± 4.0 μM (Equation (12)). Conversely, ${\mathrm{NADP}}^{+}$ functioned as an uncompetitive inhibitor toward the oxidant (at 100 μM NADPH), characterized by parallel lines in the Lineweaver–Burk plot (Figure 5B). The resulting $K_{iu}$ was to 447.4 ± 54.5 μM, as calculated according to Equation (13).

To evaluate the oxidant specificity of *Ct*FNR, we characterized a diverse array of nonphysiological electron acceptors. This includes quinones (Q), nitroaromatics (${\mathrm{ArNO}}_{2}$), aromatic *N*-oxides (ArN→O) with single-electron reduction midpoint potentials ($E_{7}^{1}$) spanning 0.09 V to −0.494 V. Additionally, single-electron acceptors such as ferricyanide, benzyl viologen (${\mathrm{BV}}^{2+}$) and ${\mathrm{Fe}\left(\mathrm{EDTA}\right)}^{-}$ were included. The calculated $k_{\mathrm{cat}}^{\mathrm{app}}$ and $k_{\mathrm{cat}}/K_{m}$ values at 100 µM NADPH are summarized in Table 1, derived from non-linear regression using Equation (9) or Equation (10). Notably, the $k_{\mathrm{cat}}/K_{m}$ for DQ closely aligns with the apparent bimolecular reaction rate constant observed in our presteady-state analysis (Table 1, Figure 3B), validating the consistency of our kinetic model.

**Figure 6:**
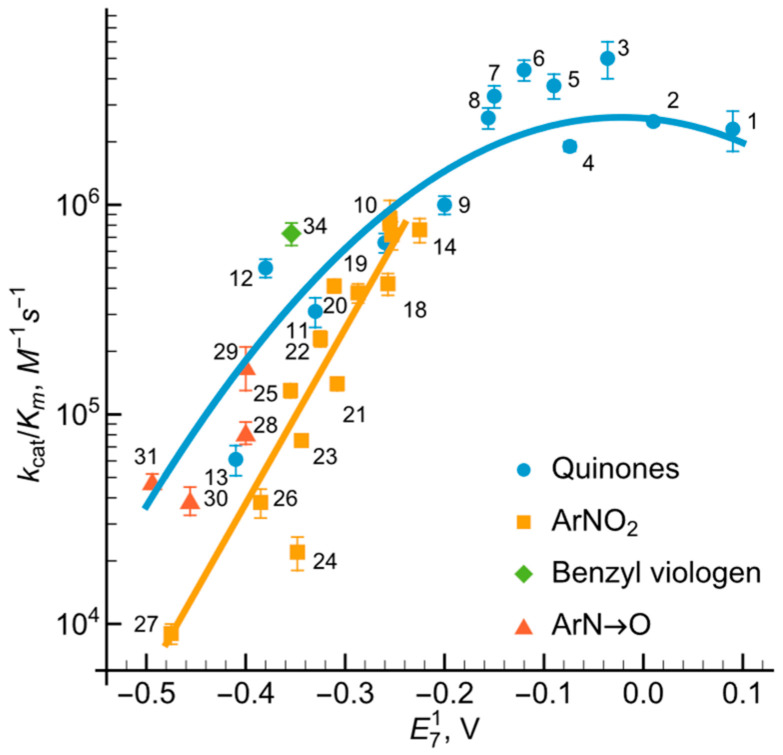
The dependence of the reactivity of quinones, nitroaromatic compounds, aromatic *N*-oxides and benzyl viologen on their single-electron reduction midpoint potentials ($\log_{10}$ scale). The compounds and their reduction potentials corresponding to the numbers are given in Table 1.

The reactivity of the examined nitroaromatics, expressed as log $k_{\mathrm{cat}}/K_{m}$, demonstrated a linear correlation with their $E_{7}^{1}$ values (Figure 6), where nifuroxime and nitrofurantoin emerged as the most efficient oxidants. In contrast, the reactivity profiles for quinones and aromatic *N*-oxides followed a parabolic dependence on $E_{7}^{1}$ with both chemical groups displaying comparable log $k_{\mathrm{cat}}/K_{m}$ magnitudes. Notably, the single-electron acceptor benzyl viologen (compound 34, Figure 6) showed a reactivity level consistent with quinones of similar reduction potentials. Collectively, these data suggest that the redox reactions are governed primarily by thermodynamic driving force ($E_{7}^{1}$) rather than specific structural recognition of the oxidant’s scaffold.

Our results demonstrate that *Ct*FNR primarily reduces quinones and nitroaromatics to their radical forms. To quantify this, we measured the single-electron flux using the cytochrome *c* reduction assay at pH < 7.2 [34]. For *Ct*FNR, the ratio of cytochrome *c* reduction to the doubled rate of NADPH oxidation was 155%, indicating a 77% single-electron flux. When 4-nitroacetophenone was employed as the oxidant, we observed a 90% single-electron flux. Interestingly, the addition of superoxide dismutase (SOD) reduced the cytochrome *c* reduction rate by 25%. This suggests that the nitroaromatic radical reacts with oxygen to generate superoxide, which subsequently contributes to cytochrome *c* reduction [35,36].

**Figure 7:**
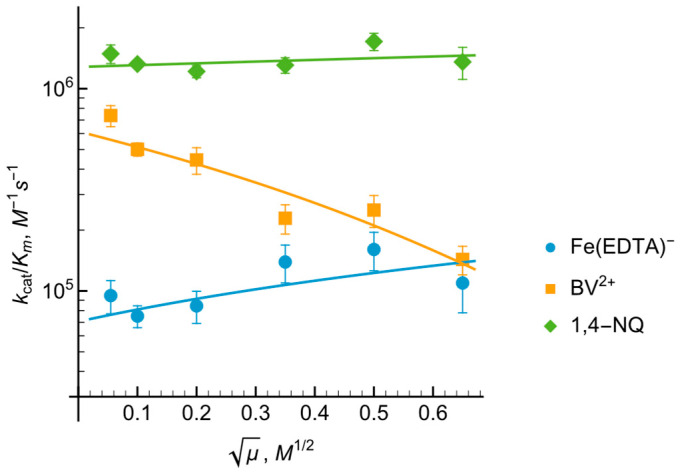
The dependence of $k_{\mathrm{cat}}/K_{m}$ (logarithmic scale) of *Ct*FNR-catalyzed reaction on the ionic strength of the buffer solution with the electron acceptors being a negatively charged ${\mathrm{Fe}\left(\mathrm{EDTA}\right)}^{-}$ (blue circles), positively charged benzyl viologen (${\mathrm{BV}}^{2+}$, orange squares) and neutral 1,4-naphthoquinone (green diamonds).

To investigate the role of electrostatic forces in *Ct*FNR reoxidation, we examined the impact of ionic strength on oxidant reactivity (Figure 7). The log $k_{\mathrm{cat}}/K_{m}$ for the cationic benzyl viologen (${\mathrm{BV}}^{2+}$) decreased as ionic strength increased, whereas the reactivity of anionic ${\mathrm{Fe}\left(\mathrm{EDTA}\right)}^{-}$ showed the opposite trend. The reactivity of neutral 1,4-naphthoquinone was largely independent of ionic strength. These observations suggest that these electorn acceptors interact with a negatively charged region of the *Ct*FNR protein surface.

### 2.3. Redox Potential Determination

Building on our previous characterization of FNRs from *R*. *palustris* and *B*. *subtilis* [21,22], we utilized the Haldane relationship to determine the standard redox potential ($E_{7}^{0}$) of the $E\text{-}\mathrm{FAD}/{E\text{-}\mathrm{FADH}}^{-}$ couple in *Ct*FNR. For two-electron hydride transfer, the potential difference between reactants is described by Equation (3):

(3) $$\Delta E^{0}\left(V\right)=0.0295\times \log K$$

**Figure 8:**
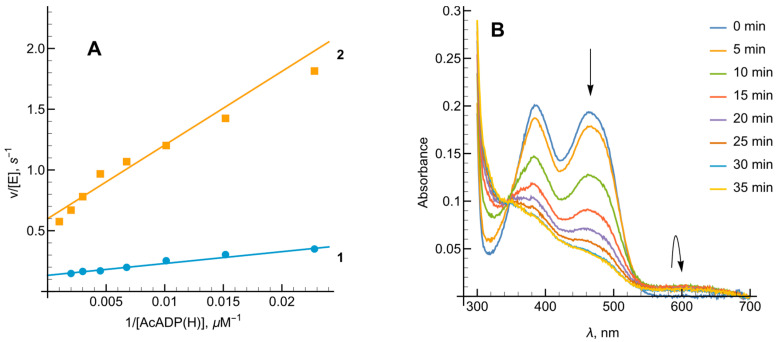
(**A**) Lineweaver–Burk plots for the transhydrogenase reaction with AcADP(H) with varied concentration of ${\mathrm{AcADP}}^{+}$ at 200 µM NADPH (line 1, blue) or varied concentration of AcADPH at 1.0 mM ferricyanide (line 2, orange). (**B**) The reduction of *Ct*FNR with a catalytic amount of NADPH under anaerobic conditions using an NADPH regeneration system at different timepoints of the reaction. Arrows indicate the disappearance of flavin peak ~460 nm and the appearance of the semiquinone at the 600 nm region.

To circumvent the kinetic complexities of ${\mathrm{NADP}}^{+}$ reduction, we employed the analog 3-acetylpyridine adenine dinucleotide phosphate (AcADP(H), $E_{7}^{0}$ = −0.258 V [37]). We monitored the forward reaction (AcADPH oxidation) by observing 1 mM ferricyanide reduction, with AcADPH generated in situ using a glucose-6-phosphate dehydrogenase system. The rate constants obtained were $k_{\mathrm{cat}}$ = 1.63 ± 0.12 $s^{-1}$ and $k_{\mathrm{cat}}/K_{m}$ = 1.6 ± 0.3 × $10^{4}$ $M^{-1}s^{-1}$ (on a two-electron basis) (Figure 8A). For the reverse reaction (reduction of ${\mathrm{AcADP}}^{+}$ by 200 µM NADPH) the constants were $k_{\mathrm{cat}}$ = 7.54 ± 0.24 $s^{-1}$ and $k_{\mathrm{cat}}/K_{m}$ = 1.0 ± 0.1 × $10^{5}$ $M^{-1}s^{-1}$. No inhibition was observed when performing the transhydrogenase reaction with constant NADPH concentrations in the range of 25–200 µM. These data yielded an equilibrium constant *K* = 0.16 ± 0.04, corresponding to a calculated $E_{7}^{0}$ of −0.282 ± 0.003 V.

To evaluate the concentration of ${\mathrm{FADH}}^{•}$ at equilibrium and determine the individual redox potentials for the two single-electron transfers, we initially followed the photoreduction methodology used for *Rp*FNR and *Bs*FNR using 5-deazaFMN and EDTA [21,22]. However, this approach was ineffective for *Ct*FNR, even at high reagent concentrations. As an alternative, we achieved enzyme-bound FAD reduction by employing a catalytic amount of NADPH (1 μM) and an enzymatic regeneration system (glucose-6-phosphate/glucose-6-phosphate dehydrogenase). Strictly anaerobic conditions were maintained using an excess of glucose, glucose oxidase and catalase to scavenge residual $O_{2}$ and $H_{2}O_{2}$. After establishing anaerobiosis, the enzyme reduction begins immediately upon the introduction of NADPH, marked by an absorbance decrease at 460 nm and increase at 600 nm, characteristic of the neutral FAD semiquinone (${\mathrm{FADH}}^{•}$) (Figure 8B). Upon reintroducing oxygen to the sample, the absorbance at 460 nm and 600 nm rapidly returned to the initial values.

Based on established extinction coefficients ($\varepsilon_{600}=5.0\;{\mathrm{mM}}^{-1}{\mathrm{cm}}^{-1}$) [38], the maximum semiquinone yield was calculated to be at 11%. By utilizing sub-stoichiometric concentration of NADPH, we minimized spectral interference from enzyme-NADP(H) complexes [18,20]. The separation of the two single-electron transfer potentials $\Delta E_{7}^{1}=E_{7}^{E\text{-}\mathrm{FAD}/{E\text{-}\mathrm{FADH}}^{\text{•}}}-E_{7}^{{E\text{-}\mathrm{FADH}}^{\text{•}}/{E\text{-}\mathrm{FADH}}^{-}}$ can then be calculated based on the semiquinone formation constant $K_{s}$ (Equations (4) and (5)):

(4) $$\frac{{\left[{E\text{-}\mathrm{FADH}}^{•}\right]}_{\max}}{{\left[E\text{-}\mathrm{FAD}\right]}_{\mathrm{tot}}}=\frac{\sqrt{K_{s}}}{2+\sqrt{K_{s}}}$$

(5) $$\Delta E_{7}^{1}\left(V\right)=0.059\times \log K_{s}$$

where ${\left[{E\text{-}\mathrm{FADH}}^{•}\right]}_{\max}$ is the maximum concentration of the semiquinone, and ${\left[E\text{-}\mathrm{FAD}\right]}_{\mathrm{tot}}$ is the total concentration of the enzyme [39]. According to our data, $K_{s}$ = 0.061 and $\Delta E_{7}^{1}$ = −0.070 V, which corresponds to $E_{7}^{E\text{-}\mathrm{FAD}/{E\text{-}\mathrm{FADH}}^{\text{•}}}$ = −0.317 V and $E_{7}^{{E\text{-}\mathrm{FADH}}^{\text{•}}/{E\text{-}\mathrm{FADH}}^{-}}$ = −0.247 V. In this case, questions may arise about the actual percentage of ${\mathrm{FADH}}^{•}$ in equilibrium, since only one of the two subunits is involved in catalysis (Figure 2A,B). However, it can be argued that the different activities of the subunits are associated with the nonequivalence of the NADP(H) binding domains, since the FAD isoalloxazine environment is the same in both subunits [12]. Moreover, the reduction of *Ct*FNR in our experiment proceeds quite slowly, and its degree of reduction significantly exceeds 50% (Figure 8B). Therefore, we believe that under these conditions a redox equilibrium is established involving both FAD-binding domains, and the resulting ${\mathrm{FADH}}^{•}$ percentage reflects identical redox properties of both subunits. On the other hand, the possibility that the obtained values reflect the average of the redox potentials of both FADs cannot be excluded; however a more detailed study of these details is a subject of our further studies.

## 3. Discussion

The results of this work, complementing our previous studies on TrxR-type *Rp*FNR and *Bs*FNR [21,22], allow us to draw more general conclusions about the catalytic mechanisms and thermodynamic properties of this relatively poorly studied group of enzymes. Although the mechanisms of reactions with nonphysiological oxidants are not necessarily analogous to reactions with physiological redox partners, they provide important information that may be valuable in the application of these enzymes.

First, as in the case of *Rp*FNR and *Bs*FNR and other types of FNRs, reoxidation of *Ct*FNR with duroquinone results in a transient 600 nm absorption (Figure 2 and Figure 3) [21,22,23]. It is consistent with a two-step reoxidation process ${\mathrm{FADH}}^{-}\to {\mathrm{FADH}}^{•}\to \mathrm{FAD}$ with ${\mathrm{FADH}}^{•}$ oxidation being rate limiting. Next, *Ct*FNR catalysis proceeds according to a “ping-pong” mechanism (Figure 4) [21,22]. Thus, it can be stated that the reduction and oxidation half-reactions occur independently, but at the same or significantly overlapping binding sites. Given that at a fixed NADPH concentration, the apparent $k_{\mathrm{cat}}$ of the reactions vary significantly with different oxidants (Table 1), this indicates that the oxidative half-reaction is rate-limiting. As in the case of *Rp*FNR and *Bs*FNR [21,22], ${\mathrm{NADP}}^{+}$ is a competitive inhibitor for NADPH and an uncompetitive inhibitor for the oxidant (Figure 5A,B). This indicates that ${\mathrm{NADP}}^{+}$ binds much more efficiently to the oxidized form of the enzyme than to the reduced form, in this case its semiquinone form. Since oxidants are likely to interact with the negatively charged domain near the isoalloxazine (Figure 7), a possible candidate is the conserved Asp64 [11]. Similar patterns have been observed in the reactions of oxidants with *Bs*FNR containing the Asp57 residue, which in turn corresponds to Asp56 in *Rp*FNR [22].

One of the most important features that unites *Ct*FNR with *Rp*FNR, *Bs*FNR and other types of FNRs is that the log $k_{\mathrm{cat}}/K_{m}$ values for quinones, aromatic nitrocompounds and *N-*oxides increase with an increase in their $E_{7}^{1}$ (Figure 6) [21,22,23], i.e., the reactivity is governed by the single-electron accepting potency of oxidants rather than by their structural features. Taken together with the dominant single-electron flux during Q and ${\mathrm{ArNO}}_{2}$ reduction, this is consistent with the “outer sphere” single electron transfer model [40,41]. In this case, the rate constant for the electron transfer between two reactants ($k_{12}$) is dependent on the electron self-exchange rate constants of those reactants ($k_{11}$ and $k_{22}$) and the reaction equilibrium constant (*K*):

(6) $$k_{12}=\sqrt{k_{11}\times k_{22}\times K\times f}$$

where log *K* is expressed as in Equation (5), and

(7) $$\log f=\frac{\log^{2}K}{4\log\left(\frac{k_{11}\;\times \;k_{22}}{Z^{2}}\right)}$$

and *Z* is the frequency factor, $10^{11}$ $M^{-1}s^{-1}$. According to Equations (5)–(7), log $k_{12}$ for a reaction of electron donor with a series of oxidants with similar $k_{22}$ values will exhibit a parabolic dependence on ${\Delta E}^{1}$, or a linear one, if ${\Delta E}^{1}$ = ±0.15 V. It has been established that the $k_{22}$ of ${\mathrm{ArNO}}_{2}$, ~$10^{6}$ $M^{-1}s^{-1}$, is 100 times lower than that of Q and ArN→O, ~$10^{8}$ $M^{-1}s^{-1}$ [42,43,44], which would lead to a 10-fold lower reactivity of ${\mathrm{ArNO}}_{2}$ when compared to quinones of similar $E_{7}^{1}$ values. A similar difference in their reactivity is observed experimentally (Figure 6).

Focusing on specific properties of *Ct*FNR, we have for the first time determined its standard redox potential of −0.282 V, which is more negative than that of *Bs*FNR (−0.240 V) and similar to that of *R. palustris* (−0.276 V) [21,22]. The similarities of the amino acids in the FAD isoalloxazine ring environment of these TrxR-type FNRs have been discussed in previous papers [21,22], therefore only the differences that may influence the standard redox potentials of the enzymes and their ${\mathrm{FADH}}^{•}$ stability will be discussed. The *si-*side of the FAD isoalloxazine of *Ct*FNR is shielded by Tyr57, along with that *Rp*FNR and *Bs*FNR have homologous Tyr49 and Tyr50, respectively [45]. In contrast, the *re*-side of the isoalloxazine of *Ct*FNR is stacked with Phe337, which corresponds toTyr328 in *Rp*FNR and His324 in *Bs*FNR [45]. The effect of the presence of His324 in *Bs*FNR is similar to the case for flavodoxin from *Desulfovibrio vulgaris*, where Tyr98His substitution increased the $E_{7}^{0}$ of FMN by 0.07 V [46]. The presence of the imidazole group of histidine is thought to stabilize the anionic form of the reduced flavin, i.e., to make its oxidation more difficult. On the other hand, the Tyr98Phe substitution has almost no effect on $E_{7}^{0}$ of flavodoxin [46]. These differences in $E_{7}^{0}$ match those observed in the aforementioned TrxR-type FNRs. It should also be noted that in the case of *Ct*FNR, the 11% stability of ${\mathrm{FADH}}^{•}$ at equilibrium is much lower than that of *Rp*FNR, 26.5%, or *Bs*FNR, 44% [21,22]. In this context, *Bs*FNR and *Rp*FNR have Thr326 and Thr330, respectively, which form H-bonds with N5 of isoalloxazine, while in *Ct*FNR these functions are performed by Ser339 [12,18,20]. It is possible that the substitution of Thr for Ser destabilizes ${\mathrm{FADH}}^{•}$, as is known in the case of neuronal NO synthase, where the Ser1176Thr mutation significantly stabilizes FAD semiquinone [47].

Some mechanistic details of the *Ct*FNR-catalyzed electron transfer can be quantitatively assessed using the data in Figure 6. According to Mauk et al., the electron transfer distance ($R_{p}$) in the reactions of metalloproteins with inorganic complexes at infinite ionic strength, i.e., in the absence of electrostatic effects, can be related to the electron self-exchange rate constant ($k_{11}$) of metalloproteins [48]:

(8) $$R_{p}\left(\text{Å}\right)=6.3-0.35\ln k_{11}$$

Based on the fact that when $\Delta E_{7}^{1}$ = 0, $k_{12}\;=\;\sqrt{k_{11}\;\times \;k_{22}}$ we have used this approach for the comparative purposes to estimate the $R_{p}$ values of various flavoenzymes dehydrogenases–electrontransferases, including the Trx-type FNRs [21,22,23]. Assuming that ${\mathrm{FADH}}^{•}$ oxidation is the rate-limiting step, we obtained that at $E_{7}^{1}$ = −0.317 V of the oxidant, the log $k_{11}$ values for *Ct*FNR are 3.6 ± 0.3 (Q), and 4.6 ± 0.4 (${\mathrm{ArNO}}_{2}$). This gives the $R_{p}$ values of 3.4 ± 0.3 Å and 2.6 ± 0.3 Å, respectively. Since it is possible that only one *Ct*FNR subunit can participate in the reaction, the value of $k_{12}$ can be multiplied by 2, and accordingly the value of $k_{11}$ can be multiplied by 4. According to Equation (8), this would further reduce the value of $R_{p}$ by 0.5 Å. One may note that these values are lower than those estimated in the cases of *Rp*FNR (5.2–5.4 Å) or *Bs*FNR (3.8 Å) [21,22]. This can be attributed either to the insufficient accuracy of Equation (8), or to the specific structural features *Ct*FNR. Recent studies have shown that the reduced FAD of *Ct*FNR is in a more viscous environment than in *Rp*FNR and *B*sFNR, and, correspondingly, contains less bound $H_{2}O$ molecules close to the active center [49]. This can decrease the local dielectric constant at the environment of isoalloxazine, which in turn may decrease the solvent reorganization energy, thus increasing an intrinsic reactivity of flavin cofactor [50,51,52]. Taken together, these data indicate that the intrinsic activity of *Ct*FNR towards nonphysiological oxidants is similar to that of *Rp*FNR and BsFNR, thus ruling out the effects of possible shielding of isoalloxazine by *C*-terminal extension [12].

In conclusion, the studies of the reactions of *Ct*FNR and other TrxR-type FNRs with nonphysiological oxidants can provide valuable information on their structural and catalytic properties, which, however, may not entirely clarify their physiologically relevant functions. On the other hand, this information may be valuable from an application perspective, including the use of anoxygenic phototrophic bacteria in photobioelectrochemical systems and microbial fuel cells [53,54,55]. In this case, a very important aspect is the ensuring of efficient electron transfer between the cellular redox enzymes and the electrode, which is carried out by redox-active mediators [56]. Our data on the efficiency of reduction of the redox mediators by flavoenzymes are directly related to this problem.

## 4. Materials and Methods

**Figure 9:**
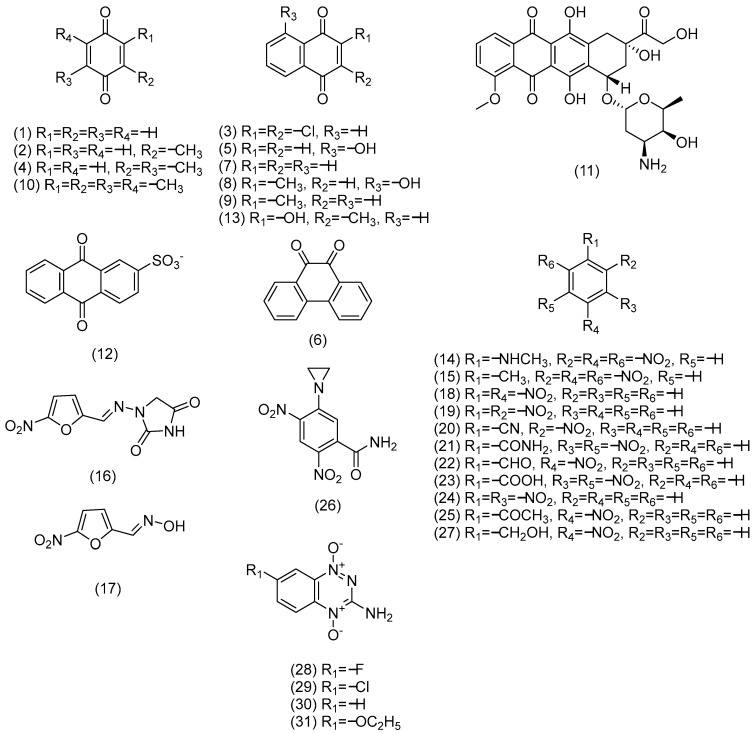
Structural formulae of nonphysiological electron-acceptors used in this study. Numbers correspond to the compounds given in Table 1.

*C. tepidum* ferredoxin:NADP^+^ oxidoreductase was purified as previously described and its concentration was determined spectrophotometrically according to $\varepsilon_{466}\;=\;10.3\;{\mathrm{mM}}^{-1}{\mathrm{cm}}^{-1}$ [12]. NADP(H), 3-acetylpyridineadenine dinucleotide phosphate (${\mathrm{AcADP}}^{+}$), horse heart cytochrome *c*, superoxide dismutase, glucose oxidase, glucose 6-phosphate, glucose 6-phosphate dehydrogenase, 5-deazaFMN and other commercially available reagents were obtained from Sigma-Aldrich (St. Louis, MO, USA) and used as received. 5-(Aziridin-1-yl)-2,4-dinitrobenzamid synthesized as described in [57], was generous gift of Dr. Vanda Miškinienė (Institute of Biochemistry, Vilnius University). 2,4,6-Trinitrotoluene and *N*-methylpicramide synthesized as described in [58,59] and tirapazamine derivatives synthesized as described in [60,61,62] were a generous gift of Dr. Jonas Šarlauskas (Institute of Biochemistry, Vilnius University). The structural formulae of nonphysiological electron acceptors are given in Figure 9:

The steady-state kinetics experiments were performed using a Cary60 UV/Vis spectrophotometer (Agilent Technologies, Santa Clara, CA, USA). All experiments were performed in 0.02 M Hepes/NaOH + 1 mM EDTA buffer solution (pH 7.0) at 25 °C. The kinetic data were fitted to the Michaelis–Menten equation in Mathematica (Wolfram Research, Inc., Mathematica, Version 14.0, Champaign, IL, USA (2024)) (Equations (9) and 10) to yield the steady-state parameters of the reactions, namely the catalytic constant $k_{\mathrm{cat}}$, bimolecular reaction rate constants (or catalytic efficiency constants) $k_{\mathrm{cat}}/K_{m}$ and substrate inhibitions constants $K_{i}$ (where applicable) of the oxidants under a constant concentration of NADPH:

(9) $$\frac{v}{\left[E\right]}=\frac{k_{\mathrm{cat}}\left[S\right]}{K_{m}+\left[S\right]}$$

(10) $$\frac{v}{\left[E\right]}=\frac{k_{\mathrm{cat}}\left[S\right]}{K_{m}+\left[S\right]\left(1+\frac{\left[S\right]}{K_{i}}\right)}$$

where v is the reaction rate, [E] is the *Ct*FNR concentration, [S] is the concentration of the oxidant and $k_{\mathrm{cat}}$ represents the number of molecules of NADPH oxidized by a single native molecule of the enzyme per second at saturated concentrations of both substrates. The fitted parameters correspond to the reciprocal intercepts and slopes of Lineweaver–Burk plots, [E]/v vs. 1/[S], respectively. The concentrations of the enzyme used in the experiments were 5–50 nM. The kinetic parameters of the steady-state reactions according to the ‘ping-pong’ mechanism were calculated according to Equation (11):

(11) $$\frac{v}{\left[E\right]}=\frac{k_{\mathrm{cat}}\left[Q\right]\left[\mathrm{NADPH}\right]}{K_{m}^{\mathrm{NADPH}}\left[Q\right]+K_{m}^{Q}\left[\mathrm{NADPH}\right]+\left[Q\right]\left[\mathrm{NADPH}\right]}$$

where Q is the electron acceptor.

The competitive inhibition constant $K_{ic}$ of ${\mathrm{NADP}}^{+}$ vs. NADPH was calculated according to Equation (12):

(12) $$\frac{v}{\left[E\right]}=\frac{k_{\mathrm{cat}}\left[Q\right]}{K_{m}^{\mathrm{NADPH}}\left(1+\frac{\left[{\mathrm{NADP}}^{+}\right]}{K_{\mathrm{ic}}}\right)+\left[\mathrm{NADPH}\right]}$$

The uncompetitive inhibition constant $K_{iu}$ of ${\mathrm{NADP}}^{+}$ vs. electron acceptor (Q) was calculated according to Equation (13):

(13) $$\frac{v}{\left[E\right]}=\frac{k_{\mathrm{cat}}\left[Q\right]}{K_{m}^{Q}+\left[Q\right]\left(1+\frac{\left[{\mathrm{NADP}}^{+}\right]}{K_{\mathrm{iu}}}\right)}$$

The rates of enzymatic NADPH oxidation in the presence of quinones, nitroaromatics, aromatic *N*-oxides or single electron acceptors were determined according to $\Delta \varepsilon_{340}\;=\;6.22\;{\mathrm{mM}}^{-1}{\mathrm{cm}}^{-1}$ and they were corrected for the intrinsic NADPH oxidase activity of *Ct*FNR (2.1 $s^{-1}$) and/or nonenzymatic NADPH oxidation by high-potential quinones. When 50 μM of cytochrome *c* was added to the reaction mixture, its quinone- and nitroaromatic-mediated reduction was assessed according to $\Delta \varepsilon_{550}\;=\;20\;{\mathrm{mM}}^{-1}{\mathrm{cm}}^{-1}$. The ferricyanide reduction rate was measured according to $\Delta \varepsilon_{420}\;=\;1.03\;{\mathrm{mM}}^{-1}{\mathrm{cm}}^{-1}$. The rate of *Ct*FNR-catalyzed reduction of ${\mathrm{AcADP}}^{+}$ by NADPH was determined according to $\Delta \varepsilon_{363}=5.6\;{\mathrm{mM}}^{-1}{\mathrm{cm}}^{-1}$ and AcADPH was prepared in situ by reducing ${\mathrm{AcADP}}^{+}$ with 10 mM glucose 6-phosphate and 0.01 mg/mL glucose 6-phosphate dehydrogenase with its concentration determined according to $\varepsilon_{363}=7.8\;{\mathrm{mM}}^{-1}{\mathrm{cm}}^{-1}$ [37]. NaCl was used to vary the ionic strength of the buffer solution. The stock solutions of organic compounds were prepared in DMSO. The final concentration of DMSO in reaction mixtures was 1% (*v*/*v*). The starting concentrations for the oxidants ranged from 100 to 1000 μM, and every compound was measured in a series of measurements with 1.5× serial dilutions for 7–10 different concentrations. The reactions were initiated by adding *Ct*FNR to the mixture of the buffer solution, NADPH and electron acceptor in the cuvette.

Presteady-state kinetics assays were performed under aerobic conditions using SX20 stopped-flow system (Applied Photophysics, Leatherhead, UK). The reduction of *Ct*FNR by NADPH and its reoxidation by a quinone or oxygen was evaluated at 460 nm and 600 nm. The *Ct*FNR in syringe 1 (~10 μM) was mixed with the contents of syringe 2 (100 μM NADPH or 100 μM NADPH + 100–500 μM of duroquinone).

The reduction of *Ct*FNR under anaerobic conditions was performed using an NADPH regeneration system with a catalytic amount of NADPH. The anaerobic cuvette contained 10 mM glucose, 50 nM glucose oxidase, 50 nM catalase, 10 mM glucose 6-phosphate, 0.01 mg/mL glucose 6-phosphate dehydrogenase and 1 μM NADPH. *Ct*FNR (~200 μM) was placed in a side arm and the cuvette was flushed with oxygen free argon for 30 min. The reaction was initiated by mixing *Ct*FNR with the cuvette contents for a final *Ct*FNR concentration of ~20 μM. The progress of the reaction was followed spectrophotometrically for 3 h.

## Figures and Tables

**Table 1:** Steady-state rate constants for the reduction of nonphysiological electron acceptors by 100 μM NADPH catalyzed by *Ct*FNR. The $E_{7}^{1}$ values taken from [25,32,33].

No.	Compound	$E_{7}^{1}$ (V)	$k_{\mathrm{cat}}^{\mathrm{app}}$$(s^{-1}$)	$k_{\mathrm{cat}}/K_{m}$$\;(M^{-1}s^{-1}$)
Quinones				
1	1,4-Benzoquinone	0.090	40.0 ± 2.5	$2.3\;\pm \;0.5\;\times \;10^{6}$
2	2-Methyl-1,4-benzoquinone	0.010	34.6 ± 0.6	$2.5\;\pm \;0.1\;\times \;10^{6}$
3	2,3-Dichloro-1,4-naphthoquinone	−0.036	42.6 ± 3.5	$5.0\;\pm \;1.0\;\times \;10^{6}$
4	2,3-Dimethyl-1,4-benzoquinone	−0.074	61.8 ± 1.2	$1.9\;\pm \;0.1\;\times \;10^{6}$
5	5-Hydroxy-1,4-naphthoquinone	−0.090	27.7 ± 0.9	$3.7\;\pm \;0.5\;\times \;10^{6}$
6	9,10-Phenanthrene quinone	−0.120	36.4 ± 6.9	$4.4\;\pm \;0.5\;\times \;10^{6}$
7	1,4-Naphthoquinone	−0.150	39.1 ± 1.4	$3.3\;\pm \;0.4\;\times \;10^{6}$
8	5-Hydroxy-2-methyl-1,4-naphthoquinone	−0.156	29.4 ± 1.5	$2.6\;\pm \;0.3\;\times \;10^{6}$
9	2-Methyl-1,4-naphthoquinone	−0.200	29.4 ± 1.3	$1.0\;\pm \;0.1\;\times \;10^{6}$
10	Tetramethyl-1,4-benzoquinone	−0.260	63.6 ± 4.3	$6.6\;\pm \;0.7\;\times \;10^{5}$
11	Doxorubicin	−0.330	25.6 ± 2.9	$3.1\;\pm \;0.5\;\times \;10^{5}$
12	9,10-Anthraquinone-2-sulphonate	−0.380	23.5 ± 0.9	$5.0\;\pm \;0.5\;\times \;10^{5}$
13	2-Hydroxy-3-methyl-1,4-naphthoquinone	−0.410	21.8 ± 1.8	$6.1\;\pm \;1.0\;\times \;10^{4}$
Nitroaromatics				
14	*N*-methylpicramide	−0.225	32.5 ± 1.0	$7.6\;\pm \;1.0\;\times \;10^{5}$
15	2,4,6-Trinitrotoluene	−0.253	41.0 ± 2.0	$7.2\;\pm \;1.1\;\times \;10^{5}$
16	Nitrofurantoin	−0.255	45.3 ± 2.4	$8.1\;\pm \;1.3\;\times \;10^{5}$
17	Nifuroxime	−0.255	68.2 ± 5.2	$8.6\;\pm \;1.9\;\times \;10^{5}$
18	*p*-Dinitrobenzene	−0.257	11.2 ± 1.3	$4.2\;\pm \;0.5\;\times \;10^{5}$
19	*o*-Dinitrobenzene	−0.287	11.0 ± 0.5	$3.8\;\pm \;0.4\;\times \;10^{5}$
20	2-Nitrobenzonitrile	−0.308	57.4 ± 2.9	$1.4\;\pm \;0.1\;\times \;10^{5}$
21	3,5-Dinitrobenzamide	−0.311	40.1 ± 1.3	$4.1\;\pm \;0.3\;\times \;10^{5}$
22	4-Nitrobenzaldehyde	−0.325	31.2 ± 2.1	$2.3\;\pm \;0.2\;\times \;10^{5}$
23	3,5-Dinitrobenzoic acid	−0.344	29.7 ± 0.5	$7.5\;\pm \;0.5\;\times \;10^{4}$
24	*m*-Dinitrobenzene	−0.348	13.6 ± 2.0	$2.2\;\pm \;0.4\;\times \;10^{4}$
25	4-Nitroacetophenone	−0.355	16.3 ± 0.7	$1.3\;\pm \;0.1\;\times \;10^{5}$
26	5-(Aziridin-1-yl)-2,4-dinitrobenzamide	−0.385	8.8 ± 0.6	$3.8\;\pm \;0.6\;\times \;10^{4}$
27	4-Nitrobenzyl alcohol	−0.475	12.9 ± 0.7	$9.0\;\pm \;1.0\;\times \;10^{3}$
Aromatic *N*-oxides				
28	7-Fluorotirapazamine	−0.400	17.4 ± 1.0	$8.2\;\pm \;1.0\;\times \;10^{4}$
29	7-Chlorotirapazamine	−0.400	14.0 ± 1.2	$1.7\;\pm \;0.2\;\times \;10^{5}$
30	Tirapazamine	−0.456	6.3 ± 0.4	$3.9\;\pm \;0.6\;\times \;10^{4}$
31	7-Ethoxytirapazamine	−0.494	16.5 ± 0.6	$4.8\;\pm \;0.4\;\times \;10^{4}$
Single electron acceptors				
32	Ferricyanide ^a^	0.410	13.6 ± 0.9	$9.2\;\pm \;1.5\;\times \;10^{4}$
33	${\mathrm{Fe}\left(\mathrm{EDTA}\right)}^{-}$	0.120	14.9 ± 1.2	$9.5\;\pm \;1.8\;\times \;10^{4}$
34	Benzyl viologen	−0.354	31.5 ± 0.8	$7.3\;\pm \;0.9\;\times \;10^{5}$

^a^—according to the rate of ferricyanide reduction.

## Data Availability

The data cited here can be provided upon reasonable request.

## Abbreviations

The following abbreviations are used in this manuscript:

${\mathrm{ArNO}}_{2}$	Nitroaromatic compound
ArN→O	Aromatic *N*-oxide
*Bs*FNR	Ferredoxin: ${\mathrm{NADP}}^{+}$ oxidoreductase from *Bacillus subtilis*
*Ct*FNR	Ferredoxin: ${\mathrm{NADP}}^{+}$ oxidoreductase from *Chlorobaculum tepidum*
DQ	Duroquinone (1,4-benzoquinone)
$E_{7}^{1}$	Single-electron reduction potential at pH 7.0
Fd	Ferredoxin
Fld	Flavodoxin
FNR	Ferredoxin: ${\mathrm{NADP}}^{+}$ oxidoreductase
NQ	1,4-naphthoquinone
Q	Quinone
*Rp*FNR	Ferredoxin: ${\mathrm{NADP}}^{+}$ oxidoreductase from *Rhodopseudomonas palustris*
TrxR	Thioredoxin reductase
